# Treatment patterns and outcomes in adolescents and young adults with Hodgkin lymphoma in pediatric versus adult centers: An IMPACT Cohort Study

**DOI:** 10.1002/cam4.3138

**Published:** 2020-05-22

**Authors:** Sumit Gupta, Nancy N. Baxter, David Hodgson, Angela Punnett, Rinku Sutradhar, Jason D. Pole, Chenthila Nagamuthu, Cindy Lau, Paul C. Nathan

**Affiliations:** ^1^ Division of Haematology/Oncology The Hospital for Sick Children Toronto ON Canada; ^2^ Faculty of Medicine University of Toronto Toronto ON Canada; ^3^ Cancer Research Program Institute for Clinical Evaluative Sciences Toronto ON Canada; ^4^ Institute for Health Policy, Evaluation and Management University of Toronto Toronto ON Canada; ^5^ Dalla Lana School of Public Health University of Toronto Toronto ON Canada; ^6^ Department of Surgery Li Ka Shing Knowledge Institute St. Michael’s Hospital Toronto ON Canada; ^7^ Princess Margaret Cancer Centre Toronto ON Canada; ^8^ Pediatric Oncology Group of Ontario Toronto ON Canada; ^9^ Center for Health Services The University of Queensland Brisbane Australia

**Keywords:** adolescents and young adults, health Services Research, Hodgkin lymphoma, population‐based, survival

## Abstract

Hodgkin lymphoma (HL) is a common adolescent and young adult (AYA) cancer. While outcome disparities between pediatric vs. adult centers [locus of care (LOC)] have been demonstrated in other AYA cancers such as acute lymphoblastic leukemia, they have not been well studied in HL. We therefore compared population‐based treatment patterns and outcomes in AYA HL by LOC. The IMPACT Cohort includes data on all Ontario, Canada AYA (15‐21 years) diagnosed with HL between 1992 and 2012. Linkage to population‐based health administrative data identified late effects. We examined LOC‐based differences in treatment modalities, cumulative doses, event‐free survival (EFS), overall survival (OS), and late effects. Among 954 AYA, 711 (74.5%) received therapy at adult centers. Pediatric center AYA experienced higher rates of radiation therapy but lower cumulative doses of doxorubicin and bleomycin. 10‐year EFS did not differ between pediatric vs. adult cancer vs. community centers (83.8% ± 2.4% vs. 82.8% ± 1.6% vs. 82.7%±3.0%; *P* = .71); LOC was not significantly associated with either EFS or OS in multivariable analyses. Higher incidences of second malignancies in pediatric center AYA and of cardiovascular events in adult center AYA were observed, but were not significant. In conclusion, while pediatric and adult centers used different treatment strategies, outcomes were equivalent. Differences in treatment exposures are however likely to result in different late‐effect risks. Protocol choice should be guided by individual late‐effect risk.

## Graphical abstract

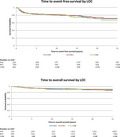

**Abbreviations**
ABVD: adriamycin, bleomycin, vinblastine, dacarbazine
ABVE‐PC: adriamycin, bleomycin, vincristine, etoposide, prednisone, cyclophosphamide
ALL: acute lymphoblastic leukemia
ALR: activity level reporting
AMI: acute myocardial infarction
AYA: adolescent and young adult
BEACOPP: bleomycin, etoposide, adriamycin, cyclophosphamide, vincristine, procarbazine, prednisolone
CABG: coronary artery bypass graft
CGy: centigray
CHF: congestive heart failure
CI: confidence interval
CIHI: Canadian Institute of Health Information
CMT: combined modality treatment
CV: cardiovascular
DAD: discharge abstract database
ED: emergency department
EFS: event‐free survival
HL: Hodgkin lymphoma
HR: hazard ratio
IMPACT: Initiative to Maximize Progress in Adolescent and Young Adult Cancer Study
IQR: interquartile range
IU: international unit
LOC: locus of care
MOHLTC: Ontario Ministry of Health and Long‐Term Care
N: number
NACRS: National Ambulatory Care Reporting System
OCR: Ontario Cancer Registry
OHIP: Ontario Health Insurance Plan claims database
OR: odds ratio
OS: overall survival
PCI: percutaneous coronary intervention
POGONIS: Pediatric Oncology Group of Ontario Networked Information System
RCC: regional cancer center
RPDB: registered persons database
RT: radiation therapy
SMN: second malignant neoplasms

## 1 INTRODUCTION

Adolescents and young adults (AYA) with cancer are an understudied group often combined with either children or older adults in clinical trials. Where pediatric and adult approaches to AYA malignancies differ, AYA patients may thus receive different treatments depending on their locus of care (LOC). In acute lymphoblastic leukemia (ALL), several studies have confirmed the superiority of pediatric protocols in AYA populations.^1^, ^2^, ^3^, ^4^

Hodgkin lymphoma (HL) is a common AYA malignancy, representing 12% of cancers among 15‐29‐year‐olds.^5^ Among patients treated on the adult studies of the German Hodgkin Study Group, the outcomes of adolescents aged 15‐20 years were similar to those aged 21‐45 years.^6^ In contrast, on recent Children's Oncology Group HL trials, event‐free survival (EFS) was inferior among AYA compared to younger children.^7^ It is still unclear however whether AYA with HL should be offered adult or pediatric protocols.^8^ Pediatric protocols have generally followed a strategy of upfront chemotherapy dose intensity in order to limit cumulative doses of chemotherapy. Whether pediatric protocols offer an advantage in either outcome or the risk of late effects is unknown.

Using a population‐based cohort of AYA in Ontario, Canada, our objective was therefore to determine whether treatment exposures and cancer outcomes differed by LOC [pediatric vs. adult regional cancer center (RCC) vs. community center]. We also determined whether the cumulative incidence of specific late effects differed by LOC.

## 2 MATERIALS AND METHODS

### 2.1 Study setting

Canadian healthcare is administered through provincial universal insurance systems in which most physicians operate on a fee‐for‐service basis. Physicians on alternative payment plans are still required to submit shadow‐billing claims. Pediatric oncology care in Ontario is delivered through five tertiary centers. Adolescents aged 15‐18 receive care at either pediatric or adult centers, while older AYA nearly always receive care in adult centers.^9^

### 2.2 IMPACT Cohort

The Initiative to Maximize Progress in Adolescent and Young Adult Cancer (IMPACT) study collected detailed patient, disease, treatment, and outcome data on all AYA aged 15‐21 years diagnosed in Ontario between 1992 and 2012 with one of six malignancy types: acute leukemia, HL, non‐Hodgkin lymphoma, soft tissue sarcoma, bone sarcoma, and testicular cancer. Details on the IMPACT Cohort methodology have been published previously.^10^ In brief, AYA treated in pediatric centers were identified through a provincial pediatric cancer registry, Pediatric Oncology Group of Ontario Networked Information System (POGONIS),^9^ while AYA treated in an adult center were identified through the Ontario Cancer Registry (OCR) and their clinical data obtained through chart abstraction. Robust protocols for real‐time data review by clinicians ensured quality abstraction. Abstracted variables included malignancy‐level data (stage and histology) and cancer events (eg, relapse, progression, and second malignancies). Pathology reports were scanned to facilitate centralized verification of findings.

Treatment variables included cancer surgeries, radiation (dose/field), chemotherapeutic, biologic and hormonal agents, and stem cell transplantation. Total dose (per m^2^) calculated for chemotherapies was mostly associated with late effects (eg, anthracyclines, bleomycin, alkylating agents). Demographic data were obtained from the Registered Persons Database (RPDB), a provincial vital statistics registry. Death (from RPDB) and second malignancies (from OCR) were confirmed by chart abstraction. Records prior to death were reviewed to attribute cause of death.

### 2.3 Additional data sources

Patients were linked deterministically to population‐based health services databases housed at ICES, a research institute encompassing an array of Ontario health‐related data. These datasets were linked using uniquely encoded identifiers and analyzed at ICES. These health services databases allowed the identification of hospitalizations, emergency room visits, and physician encounters (Table S1).

### 2.4 Outcomes

The primary outcomes were event‐free and overall survival (EFS, OS), both measured from the time of initial diagnosis. Events included relapse, progressive disease, death, and subsequent malignancy. Validated algorithms, described in detail previously, using health services data were used to identify late cancer events.^11^ In brief, this involved identifying billings for chemotherapy, radiation, or palliative care with service dates after the end of the initial line of therapy to identify late relapses or second malignancies. Investigators reviewed patterns of healthcare use around each algorithm‐identified event to ensure validity.

Secondary outcomes included specific late effects known to account for a significant morbidity and mortality among patients with HL: second malignant neoplasms (SMN), congestive heart failure (CHF), and other major cardiovascular events. SMN were captured through the OCR and verified through chart abstraction or review of health administrative data. Congestive heart failure and major cardiovascular events (composite outcome consisting of CHF, acute myocardial infarction, stroke, percutaneous coronary intervention, and coronary artery bypass graft surgery) were identified using previously validated algorithms (Appendix A).^12^, ^13^ Infertility is also a significant concern among AYA with cancer.^14^ As a proxy for infertility, liveborn births were therefore identified through MOMBABY, a population‐based database linking the hospital admission records of delivering mothers and newborns.^15^, ^16^, ^17^

### 2.5 Variables

LOC was categorized as pediatric versus adult center based on the institution delivering the majority of the first three months of therapy. Adult centers were further categorized as RCC, as designated by Cancer Care Ontario, or community hospitals. While data on exact treatment protocol were not consistently available, during the study period pediatric centers only used pediatric protocols for HL, predominantly those developed by the Children's Oncology Group (COG) or its predecessors, or by the German Society of Pediatric Oncology and Hematology.^18^, ^19^, ^20^ These pediatric protocols were not used in adult centers, which used predominantly adriamycin, bleomycin, vinblastine, and dacarbazine (ABVD)‐based regimens.

Other patient‐level predictors included age at diagnosis and sex. Neighborhood income quintile and urban/rural status were determined using data from the Canadian census.^21^, ^22^ Time period of diagnosis was defined as early (1992‐1998), middle (1999‐2005), or late (2006‐2012). Disease‐level variables included histology and Ann Arbor stage. Patients were also categorized as having either limited [Ann Arbor IA or IIA with no bulk (<10 cm)] versus advanced (all others) disease.

### 2.6 Analyses

Patient, disease, and treatment variables were compared across LOC using chi‐squared tests or Fisher's exact tests for categorical variables as appropriate, and t‐tests for continuous variables. Trends in the annual rate of combined modality treatment (ie, both chemotherapy and radiation (CMT)) were examined over time using Poisson regression. The number of eligible patients in each year was the denominator, the natural logarithm of which functioned as an offset variable. Time period was incorporated as a covariate. Changes in cumulative chemotherapy and radiation doses over time were determined using linear regression models. Predictors of CMT were determined using logistic regression models. EFS and OS distributions were estimated using the Kaplan–Meier approach, and compared between LOC with the log rank test. Predictors of EFS and OS were determined using univariate and multivariable Cox Proportional Hazards regression models. In all regression models, predictors significant at the *P* < .1 level in univariate analyses were included in multivariable models, though as a key predictor of interest, LOC was retained regardless of univariate significance. The cumulative incidence function approach was used to determine the risk of various late effects and the incidence of liveborn births; these risks were compared by LOC using Gray's test. Death was considered a competing event. Statistical significance was defined as *P* < .05. Analyses were performed using SAS, version 9.4 (SAS Institute). Ethics approval was obtained at The Hospital for Sick Children, St. Michael's Hospital, and Sunnybrook Hospital. Informed consent was not required.

## 3 RESULTS

Over the study period, 954 AYA were diagnosed with HL, 711 (74.5%) of whom were treated in adult centers. Median follow‐up was 6 years (range 0‐25). Demographic and disease characteristics, stratified by LOC, are shown in Table 1. As compared to AYA at adult centers, pediatric center AYA were younger and more likely to be treated in the later time period. They were less likely to have mixed cellularity histology, and more likely to have Stage IV disease (Table 1). There was no difference in the presence of B symptoms. Pediatric center AYA were also more likely to be registered on clinical trials [95/243 (39.1%) vs. 27/706 (3.8%); *P* < .001].

**TABLE 1:** Demographic and disease characteristics of study cohort (N = 954), stratified by locus of care

	Pediatric center (N = 243)	Adult RCC (N = 550)	Community center (N = 161)	*P* value
Age (years, median, IQR)	16 (15‐17)	19 (18‐20)	19 (18‐20)	**<.001**
Sex (N, %)				.67
Male	119 (49.0)	255 (46.4)	80 (49.7)	
Female	124 (51.0)	295 (53.6)	81 (50.3)	
Time period (N, %)				**<.001**
Early (1992‐1998)	54 (22.2)	214 (38.9)	46 (28.6)	
Middle (1999‐2005)	82 (33.7)	185 (33.6)	57 (35.4)	
Late (2006‐2011)	107 (44.0)	151 (27.5)	58 (36.0)	
Neighborhood income quintile (N, %)				.12
Q1 (lowest)	31 (12.9)	104 (19.0)	27 (16.9)	
Q2	46 (19.1)	108 (19.8)	26 (16.3)	
Q3	46 (19.1)	96 (17.6)	43 (26.8)	
Q4	55 (22.8)	121 (22.2)	33 (20.6)	
Q5 (highest)	63 (26.1)	117 (21.4)	31 (19.4)	
Rurality (N, %)				.24
Urban	213 (87.7)	481 (87.9)	131 (81.9)	
Rural	30 (12.3)	66 (12.1)	29 (18.1)	
Histology (N, %)				**<.001**
Nodular sclerosis	209 (86.0)	474 (86.2)	137 (85.1)	
Mixed cellularity	12 (4.9)	52 (9.5)	15 (9.3)	
Lymphocyte deplete	<=5^a^	<=5^a^	<=5^a^	
Lymphocyte rich	19 (7.8)	<=10	<=5^a^	
Other	<=5^a^	11 (2.0)	<=5^a^	
Stage (N, %)				**<.001**
I	10 (4.5)	51 (9.3)	15 (9.3)	
II	128 (57.9)	356 (65.0)	98 (60.9)	
III	35 (15.8)	88 (16.1)	30 (18.6)	
IV	48 (21.7)	53 (9.7)	18 (11.2)	
B symptoms (N, %)				.07
No	123 (55.7)	337 (61.5)	108 (67.1)	
Yes	98 (44.3)	211 (38.5)	53 (32.9)	
Disease extent (N, %)				.60
Limited	90 (40.7)	245 (44.7)	71 (44.1)	
Advanced	131 (59.3)	303 (55.3)	90 (55.9)	

Note

Bold signifies statistical significance. Abbreviations: IQR, interquartile range; N, number; RCC, regional cancer center.

^a^ Privacy regulations prevent the disclosure of small cell sizes ≤ 5.

Presence or absence of bulk disease was available on 548/711 (77.1%) of adult center AYA but only on 11/243 (4.5%) pediatric center AYA. Pediatric center AYA with known vs. unknown bulk disease were not significantly different in age at diagnosis, sex, histology, or Ann Arbor stage, but were more likely to have been diagnosed in the late time period (data not shown). Analyses revealed that of adult center AYA with Stage IA or IIA disease, only 27/188 (14.4%) were noted to have bulk disease. Pediatric patients with Stage IA or IIA disease with unknown disease bulk were therefore subsequently categorized as limited stage.

AYA treated at pediatric centers were significantly more likely to receive radiation therapy (RT) (182/243, 74.9%) vs. those at adult centers (RCC—288/550, 52.4%; community center—83/161, 51.6%; *P* < .001) although, among those receiving RT, the median radiation dose was higher at adult centers (30 and 35 Gy at community and RCC vs. 21 Gy at pediatric centers; *P* < .001). Cumulative doses of doxorubicin were significantly higher at adult centers (median cumulative doses 297 mg/m^2^ vs. 291 mg/m^2^ vs. 200 mg/m^2^ in community, RCC and pediatric centers, respectively, *P* = .02). Similarly, bleomycin doses were also higher at adult compared to pediatric centers (Table 2).

**TABLE 2:** Treatment modalities and intensity by locus of care among adolescents and young adults with Hodgkin lymphoma

	Pediatric center (N = 243)	Adult regional cancer center (N = 550)	Adult community center (N = 161)	*P* value
Patients receiving combined modality treatment [N (%)]	182 (74.9%)	288 (52.4%)	83 (51.6%)	<.001
Patients receiving radiation [N (%)]	183 (76.0%)	265 (49.6%)	78 (48.4%)	<.001
Radiation dose [Gray, Median (IQR)]	21 (21‐21)	35 (30‐35)	30 (25‐35)	<.001
Cumulative doxorubicin dose [mg/m^2^, Median (IQR)]	200 (170‐232)	291 (201‐303)	297 (203‐304)	.02
Cumulative bleomycin dose [IU/m^2^, Median (IQR)]	60 (40‐60)	114 (78‐121)	114 (78‐121)	<.001

Note

Abbreviations: IQR, interquartile range; IU, international unit; N, number.

A small but statistically significant decrease in radiation dose, driven predominantly by patients with limited stage disease and in RCC, was seen over the study period (Table 3). Similarly, a small but significant increase in cumulative anthracycline dose, driven by patients with advanced‐stage disease, was also seen. No statistically significant change in the percentage of patients receiving CMT was seen (Table 3).

**TABLE 3:** Average annual change in treatment intensity over time

	Overall	Limited stage	Advanced stage	Pediatric centers	RCC	Community centers
Percentage receiving CMT (%)	0.0 (−0.7 to 0.7; 0.29	1.2 (−1.0 to 3.6; 0.27)	0.7 (−1.3 to 2.8; 0.50)	−1.4 (−3.7 to 0.9; 0.24)	0.9 (−1.3 to 3.1; 0.42)	0.9 (−3.0 to 5.0; 0.65)
Radiation dose (CGy)	−25 (−41 to −11; 0.003)	−20 (−35 to −5; 0.02)	−25 (−54 to 5; 0.12)	12 (−17 to 44; 0.41)	−33 (−48 to −18; 0.0005)	−12 (−49 to −26; 0.55)
Anthracycline dose (mg/m^2^)	1.2 (0.3 to 2.1; 0.02)	0.1 (−2.0 to 2.1; 0.96)	2.0 (0.7 to 3.2; 0.006)	0.4 (−1.8 to 2.6; 0.93)	1.0 (−0.03 to 2.0; 0.06)	1.2 (−1.1 to 3.4; 0.31)
Bleomycin dose (IU/m^2^)	−0.1 (−0.6 to 0.5; 0.84)	0.04 (−0.9 to 0.9; 0.93)	−0.3 (−0.9 to 0.3; 0.38)	0.2 (−0.6 to 1.1; 0.63)	0.3 (−0.2 to 0.7; 0.30)	0.7 (−0.4 to 1.9; 0.21)

Note

Statistically significant changes are colored red for increases and blue for decreases, in the following format: Average annual change (95% confidence interval; p value)

Abbreviations: CGy, Centigray; CMT, combined modality treatment; RCC, regional cancer center.

In multivariate analysis, AYA with higher stage and with B symptoms were more likely to receive chemotherapy alone versus CMT (Table 4). Even adjusting for stage, B symptoms, and histology, AYA at adult centers were still significantly more likely to receive chemotherapy as their sole treatment modality [RCC—odds ratio (OR) 5.0, 95 CI 3.0‐8.4; Community center—OR 3.9, 95 CI 2.2‐7.0; *P* < .0001] than AYA at pediatric centers. This remained true when analyses were stratified by limited vs. advanced disease (data not shown). Sensitivity analyses in which histology and stage were added to the multivariable model did not change the results (Tables S2 and S3).

**TABLE 4:** Univariate and multivariable predictors of chemotherapy alone vs. combined modality treatment (chemotherapy and radiation)

	Univariate		Multivariable	
	OR (95 CI)	*P* value	OR (95 CI)	*P* value
Age (per year)	**1.2 (1.1‐1.3)**	**<.0001**	1.0 (0.9‐1.1)	.93
Sex				
Male	Ref	Ref	—	—
Female	1.1 (0.8‐1.4)	.56	—	—
Time period				
Early (1992‐1998)	Ref	Ref	—	—
Middle (1999‐2005)	0.8 (0.6‐1.1)	.19	—	—
Late (2006‐2011)	1.0 (0.7‐1.4)	.97	—	—
Neighborhood income quintile				
Q1 (lowest)	1.1 (0.7‐1.7)	.63	—	—
Q2	1.0 (0.7‐1.6)	.89	—	—
Q3	0.9 (0.6‐1.4)	.64	—	—
Q4	0.8 (0.5‐1.2)	.22	—	—
Q5 (highest)	Ref	Ref	—	—
Rurality				
Urban	Ref	Ref	—	—
Rural	1.3 (0.8‐1.9)	.25	—	—
Histology				
Nodular sclerosis	Ref	Ref	Ref	Ref
Mixed cellularity	**1.8 (1.1‐2.9)**	**.03**	1.5 (0.9‐2.6)	.11
Lymphocyte deplete	0.5 (0.1‐2.6)	.51	0.5 (0.1‐2.6)	.37
Lymphocyte rich	0.8 (0.3‐1.7)	.39	1.2 (0.5‐3.2)	.70
Other	2.7 (0.9‐7.9)	.07	1.8 (0.6‐5.5)	.33
Stage				
I	Ref	Ref	Ref	Ref
II	1.3 (0.8‐2.4)	.30	1.4 (0.8‐2.6)	.22
III	**3.8 (2.0‐7.2)**	**<.0001**	**4.3 (2.2‐8.3)**	**<.0001**
IV	**2.9 (1.5‐5.6)**	**.001**	**4.3 (2.1‐8.8)**	**<.0001**
B symptoms				
No	Ref	Ref	Ref	Ref
Yes	**1.6 (1.2‐2.1)**	**.0009**	**1.4 (1.0‐1.9)**	**.04**
Locus of care				
Pediatric	Ref	Ref	Ref	Ref
RCC	**3.9 (2.8‐5.5)**	**<.0001**	**5.0 (3.0‐8.4)**	**<.0001**
Adult community center	**3.1 (2.0‐4.8)**	**<.0001**	**3.9 (2.2‐7.0)**	**<.0001**

Note

Bold signifies statistical significance. Abbreviations: CI, confidence interval; OR, odds ratio; RCC, regional cancer center.

**FIGURE 1:**
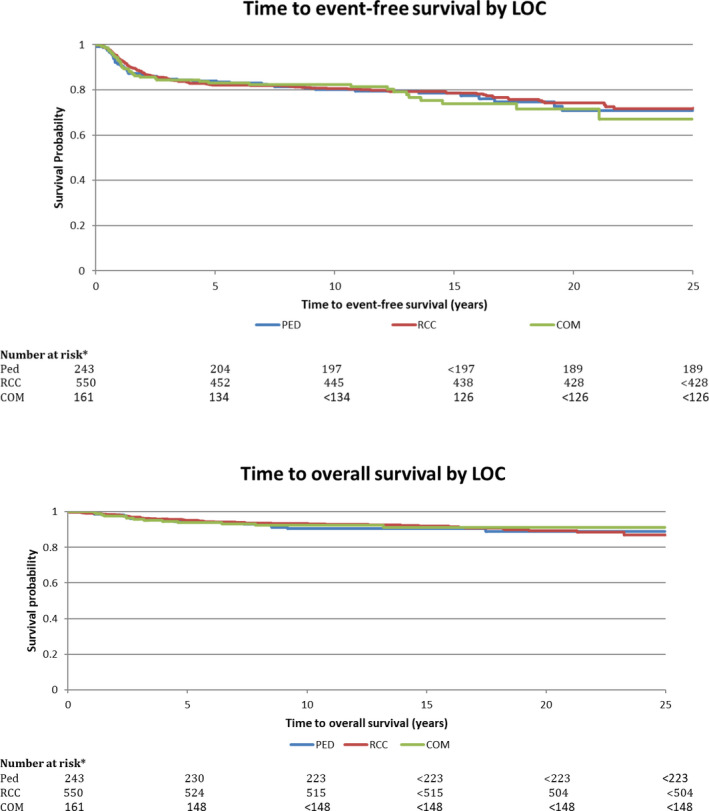
Event‐free and overall survival of adolescents and young adults with Hodgkin lymphoma by locus of care. COM, community adult center; PED, Pediatric center; RCC, regional cancer center

The 5‐year EFS and OS for the entire cohort were 83.0% ± 1.2% and 94.9% ± 0.7%, respectively. 10‐year EFS and OS were 81.1% ± 1.3% and 92.6% ± 0.7%, respectively. Neither EFS nor OS differed between AYA treated at pediatric vs. RCC vs. community centers (*P* = .71 and .90, respectively; Figure 1). In multivariable analysis, LOC was not associated with either EFS or OS (Tables 5 and 6). Sensitivity analyses stratified by limited vs. advanced disease showed similar results (data not shown).

**TABLE 5:** Univariate and multivariable predictors of event‐free survival

	Univariate		Multivariable	
	HR (95 CI)	*P* value	HR (95 CI)	*P* value
Age (per year)	1.0 (1.0‐1.1)	.25	—	—
Sex				
Male	Ref	Ref	—	—
Female	0.9 (0.7‐1.2)	.46	—	—
Time period				
Early (1992‐1998)	Ref	Ref	—	—
Middle (1999‐2005)	1.0 (0.7‐1.4)	.85	—	—
Late (2006‐2011)	1.0 (0.7‐1.5)	.89	—	—
Neighborhood income quintile				
Q1 (lowest)	1.0 (0.6‐1.5)	.87	—	—
Q2	0.8 (0.6‐1.3)	.40	—	—
Q3	0.7 (0.5‐1.1)	.13	—	—
Q4	0.7 (0.5‐1.1)	.16	—	—
Q5 (highest)	Ref	Ref	—	—
Rurality				
Urban	Ref	Ref	—	—
Rural	0.9 (0.6‐1.3)	.51	—	—
Histology				
Nodular sclerosis	Ref	Ref	Ref	Ref
Other	**0.6 (0.4‐1.0)**	**.05**	**0.6 (0.3‐0.9)**	**.02**
Stage				
I	Ref	Ref	—	—
II	1.1 (0.6‐2.0)	.69	—	—
III	1.3 (0.7‐2.5)	.36	—	—
IV	1.6 (0.8‐3.1)	.15	—	—
B symptoms				
No	Ref	Ref	Ref	Ref
Yes	**1.6 (1.2‐2.1)**	**.0007**	**1.8 (1.3‐2.4)**	**.0001**
Treatment modality				
Chemotherapy only	Ref	Ref	Ref	Ref
Radiation only	**1.6 (1.0‐2.5)**	**.05**	**2.1 (1.3‐3.4)**	**.003**
Combined modality	**0.7 (0.5‐1.0)**	**.03**	**0.7 (0.5‐0.9)**	**.02**
Locus of care				
Pediatric	Ref	Ref	Ref	Ref
RCC	0.9 (0.7‐1.3)	.59	0.8 (0.6‐1.2)	.24
Adult community center	1.0 (0.7‐1.6)	.84	1.1 (0.7‐1.7)	.69

Note

Bold signifies statistical significance. Abbreviations: HR, hazard ratio; RCC, regional cancer center.

**TABLE 6:** Univariate and multivariable predictors of overall survival

	Univariate		Multivariable	
	HR (95 CI)	*P* value	HR (95 CI)	*P* value
Age (per year)	1.0 (0.9‐1.1)	.63	—	—
Sex				
Male	Ref	Ref	—	—
Female	0.7 (0.5‐1.1)	.12	—	—
Time period				
Early (1992‐1998)	Ref	Ref	—	—
Middle (1999‐2005)	1.1 (0.6‐1.7)	.84	—	—
Late (2006‐2011)	0.7 (0.4‐1.4)	.41	—	—
Neighborhood income quintile				
Q1 (lowest)	0.8 (0.2‐1.5)	.48	1.0 (0.5‐1.9)	.94
Q2	0.7 (0.4‐1.3)	.28	0.9 (0.5‐1.7)	.71
Q3	0.5 (0.2‐1.0)	.05	0.6 (0.3‐1.2)	.14
Q4	0.5 (0.3‐1.0)	.05	0.6 (0.3‐1.2)	.12
Q5 (highest)	Ref	Ref	Ref	Ref
Rurality				
Urban	Ref	Ref	—	—
Rural	1.3 (0.7‐2.3)	.39	—	—
Histology				
Nodular sclerosis	Ref	Ref	—	—
Other	1.2 (0.7‐2.2)	.48	—	—
Stage				
I	Ref	Ref	—	—
II	0.9 (0.4‐2.4)	.9	—	—
III	1.6 (0.6‐4.4)	.33	—	—
IV	1.8 (0.6‐4.9)	.28	—	—
B symptoms				
No	Ref	Ref	Ref	Ref
Yes	**2.2 (1.4‐3.5)**	**.0008**	**2.2 (1.4‐3.4)**	**.001**
Treatment modality				
Chemotherapy only	Ref	Ref	—	—
Radiation only	0.8 (0.4‐2.0)	.67	—	—
Combined modality	0.8 (0.5‐1.2)	.23	—	—
Locus of care				
Pediatric	Ref	Ref	Ref	Ref
RCC	0.9 (0.5‐1.5)	.65	1.1 (0.6‐1.9)	.76
Adult community center	0.9 (0.5‐1.9)	.84	1.2 (0.6‐2.6)	.58

Note

Bold signifies statistical significance. Abbreviations: CI, confidence interval; HR, hazard ratio; RCC, regional cancer center.

There were no significant differences in the incidence of late effects or of liveborn births by locus of care (Table S4). A higher incidence of SMNs was seen in AYA treated in pediatric centers (25‐year cumulative incidence 8.4% ± 3.1% vs. 5.7% ± 1.2%; *P* = .12), with breast cancer the most common SMN. A higher incidence of major cardiovascular (CV) events was observed in AYA treated in adult centers (25‐year cumulative incidence 2.5% ± 0.7% vs. 0.7% ± 0.7%; *P* = .10), though these differences were not statistically significant.

## 4 DISCUSSION

In this population‐based study of AYA with HL that linked registry, clinical, and health services data, we found that while treatment exposures differed significantly between those treated at pediatric versus adult institutions, outcomes were similar.

Significant treatment variation has been demonstrated in both adults and children with HL. For example, among American patients with early‐stage HL, treatment selection (CMT vs. chemotherapy alone) was influenced by age, sex, race, insurance status, and geography.^23^ Academic centers were slightly less likely to use CMT as compared to community centers. In an American pediatric cohort, the use of CMT was associated with younger age, male sex, and having private health insurance; no results by hospital type were presented.^24^ Interestingly, in our cohort of Canadian AYA, neither age, sex, income quintile, nor rurality was significantly associated with treatment selection in multivariable analysis, possibly reflecting the impact of universal health insurance and decreased barriers to care.

Indeed, in our study treatment modalities and intensity were determined primarily by the LOC, and not by patient factors. For example, rates of radiation administration were nearly double in pediatric centers than in adult centers; when adjusting for stage and B symptoms, pediatric center AYA were 5 times as likely to receive CMT. In contrast, AYA at adult centers were exposed to higher cumulative doses of both doxorubicin and bleomycin.

These differences have been noted before and reflect divergent treatment strategies and evidence bases between pediatric and adult oncologists.^19^, ^25^, ^26^ In advanced‐stage HL, for example, a review of the adult literature concluded that there was “no survival advantage in patients consolidated with… radiation as part of primary treatment.”^27^ A contemporaneous review of the pediatric literature however concluded that “radiation therapy may be crucial in high‐risk patients.”^28^ These discrepant conclusions are likely due to the different cumulative doses of chemotherapy utilized in adult vs. pediatric protocols, with the latter reluctant, for example, to use greater than 250 mg/m^2^ of doxorubicin. High cure rates in advanced HL may only be possible with either higher cumulative chemotherapy doses or the addition of radiation therapy. These differing approaches are seen in our cohort; AYA at pediatric protocols were substantially more likely to receive radiation despite higher rates of advanced disease. In early‐stage HL, both adult and pediatric trials have studied omitting radiation for patients with no other risk factors and/or good response to chemotherapy, with varying degrees of success.^20^, ^29^, ^30^, ^31^

Our results indicate that despite the differences in treatment strategies between pediatric and adult centers, both resulted in equivalent EFS and OS. This contradicts recently published findings by Henderson et al, who conducted a retrospective comparison of AYA with intermediate risk disease treated on the adult E2496 study versus on the pediatric AHOD0031 study. On E2496, patients were randomized between ABVD or Stanford V (doxorubicin, vinblastine, nitrogen mustard, etoposide, vincristine, bleomycine, prednisone) regimens. Interestingly, 65.8% of E2496 patients also received radiation. AHOD0031 patients predominately received cycles of ABVE‐PC chemotherapy (Adriamycin, bleomycin, vincristine, etoposide, prednisone, cyclophosphamide); 76.2% of patients received radiation. Both 5‐year EFS and OS were statistically significantly superior among patients on AHOD0031, though this was not observed among patients with Stage I/II disease without anemia.^32^ Risk adjustment using propensity scores did not change the results. Reasons for the discrepancy with our results are unclear. The Henderson data pertain to patients enrolled on clinical trials, who may differ from the general AYA population. A higher proportion of E2496 patients received radiation than seen in our adult center AYA. It is also possible that while adult and pediatric strategies result in equivalent outcomes overall, certain subgroups may derive more benefit from a particular treatment regimen.

While pediatric and adult treatment strategies may not result in discrepancies in EFS or OS, they may be associated with different risks of late effects. The overall burden of late effects in survivors of HL is substantial; a prior study found that the cumulative incidence of grade 3‐5 chronic conditions among children treated for HL between 1990 and 1999 was 17.5%.^33^ In the St. Jude Lifetime Cohort, by age 50, the cumulative incidence of survivors experiencing at least one grade 3‐5 cardiovascular condition was 45.5%, with a cumulative burden of 100.8 such conditions per 100 individuals.^34^ While more recently treated cohorts may experience a lower risk of effects,^33^ other studies have shown no decrease in burden.^35^ The risk of late effects is determined in large part by the cancer‐directed therapy received. For example, radiation is a significant risk factor for the development of SMNs, while both radiation and anthracyclines contribute to CV events.^34^, ^35^, ^36^, ^37^ We did not observe statistically significant differences in the cumulative incidence of various late effects between AYA treated in pediatric versus adult centers. However, pediatric center AYA (who were more likely to receive radiation) experienced a trend toward higher rates of SMNs, while adult center AYA (who received higher cumulative doses of anthracycline) experienced a trend toward higher rates of CV events. It is plausible that with a longer follow‐up more substantial differences in the incidence of various late effects will be seen.

Our results have important implications for the clinical management of this population. In a recent review, Flerlage et al stated that it was “inexcusable for AYA patients to be provided only one choice of therapy driven by the location of their first presentation.”^8^ Our data support this statement by showing equivalency in survival between pediatric and adult treatment strategies. Knowing this, the choice of regimen should ideally be based on both individualized risks of late effects (eg, sex, disease site, genetic predisposition) and the patient's specific values and preferences.^38^ This however requires that treating oncologists be familiar with both pediatric and adult treatment regimens, perhaps best accomplished through the establishment of teams with specialized AYA expertise as has already occurred in some jurisdictions.^39^, ^40^

Study strengths include the collection of detailed clinical data on a large population‐based cohort of AYA with HL. Chart abstraction was accompanied by real‐time validation by clinical experts. Linkages to health administrative data allowed for the identification of additional cancer events as well as late effects through validated algorithms. Several limitations also merit mention. First, we cannot rule out that specific subgroups of AYA with HL, defined by initial risk factors or by response, benefit more from one treatment strategy versus the other. Relatedly, while we were able to adjust our analyses for important risk factors, not all the components of HL prognostic scores such as the International Prognostic Score were available. Second, current treatment strategies continue to evolve. Since our study period, treatment regimens have included newer radiation techniques limiting fields in sites in high‐risk patients in pediatric protocols, and a decrease in both number of chemotherapy cycles and radiation dose for adults with early‐stage favorable disease.^19^, ^29^, ^30^, ^31^ These changes may well be expected to mitigate the long‐term risk of various late effects compared to older regimens. Another example pertains to the incorporation of PET‐determined response, for which public funding in Ontario began in 2009. Thus only a small percentage of our study cohort would have received PET scans, either as part of initial staging or response assessment. Third, our results may not be generalizable to settings without universal health insurance, as uninsured status has been associated with AYA HL outcome disparities.^41^ Finally, though ABVD‐based regimens remain standard in most North American adult institutions, our results may not be generalizable to more intensive BEACOPP‐based protocols (bleomycin, etoposide, Adriamycin, cyclophosphamide, vincristine, procarbazine, prednisolone), which may be advantageous in advanced adult HL.^42^, ^43^

In conclusion, while pediatric and adult centers used different strategies to treat AYA with HL, both EFS and OS were equivalent and no LOC‐based outcome disparities were observed. Differences in treatment exposures are likely however to result in different long‐term profiles of late effects. While in current practice, the decision on what regimen to use is driven primarily by locus of care, protocol choice should instead be individualized according to personal late‐effect risk and patient preferences. Collaborative efforts between pediatric and adult trial groups are also encouraged.

## BRIEF DESCRIPTION

Adolescents and young adults (AYA) with Hodgkin lymphoma (HL) are treated using both adult and pediatric protocols. We used linked clinical and health administrative data to study treatment patterns and outcomes in AYA HL between adult and pediatric centers, the first population‐based analysis of this question. We found that while pediatric and adult centers used different treatment strategies, outcomes were equivalent. Differences in treatment exposures are however likely to result in different late‐effect risks.

## CONFLICT OF INTEREST

The authors declare no competing financial interests.

## AUTHORS’ CONTRIBUTION

Contribution: SG, NB, JP, and PN conceived of and constructed the IMPACT Cohort. SG, NB, JP, PN, and RS conceived of the initial study idea. CL, CN, and RS executed the analyses. All authors were involved in the interpretation of the results. SG drafted the manuscript; all authors contributed to the writing and final draft.

## Supporting information

Table S1‐S4

## Data Availability

The study utilizes health administrative data, which contains personal health information. Privacy legislation in Ontario, Canada prevents these data from being made publicly available.

## Appendix A

### ALGORITHMS USED TO IDENTIFY CARDIAC EVENTS USING HEALTH ADMINISTRATIVE DATA

#### A1 Congestive heart failure

‐ One CIHI claim (DAD or SDS) with any of the following ICD‐10‐CA or ICD‐9‐CM codes between index date (date of diagnosis) to end of follow‐up (December 31st, 2016):
  • ICD‐10‐CA: I50 [narrow definition]
  • ICD‐10‐CA: I42, I43, 150, I51.4, J81 [broad analysis]
  • ICD‐9:428 [narrow definition]
  • ICD‐9:422, 425, 428, 518.4 [broad analysis]

OR

‐ One OHIP claim/NACRS ED record with a CHF diagnosis + a 2nd claim/record (OHIP/NACRS) for CHF diagnosis within 1 year

#### A2 Major cardiovascular event

A composite outcome of any hospitalization/ED visit for acute myocardial infarction (AMI), stroke, percutaneous coronary intervention (PCI), coronary artery bypass graft (CABG), or death from ischemic heart disease or cerebrovascular disease.

‐ Flag history of hospitalization for AMI, stroke, CHF, or history of PCI/CABG using the following diagnosis, procedure/intervention codes:
  • AMI
    ∎ ICD‐10‐CA: I21, I22
    ∎ ICD‐9:410
  • Stroke
    ∎ ICD‐10‐CA: I60, I61, I64, H341, I630, I631, I632, I633, I634, I635, I637, I638, I639
    ∎ ICD‐9:430, 431, 434, 436, 362.3
  • CHF
    ∎ ICD‐10‐CA: I50
    ∎ ICD‐9:428
  • PCI
    ∎ Intervention codes (ICD‐10‐CA): 1IJ50, 1IJ57GQ, 1IJ54
    ∎ Procedure codes (ICD‐9): 4802, 4803
  • CABG
    ∎ Intervention codes (ICD‐10‐CA): 1IJ76
    ∎ Procedure codes (ICD‐9): 481
