# Supplementary material for: Treatment patterns and outcomes in adolescents and young adults with Hodgkin lymphoma in pediatric versus adult centers: An IMPACT Cohort Study
Source: Cancer Med. 2020 May 22;9(19):6933–45. doi: 10.1002/cam4.3138 (PMC7541154; doi:10.1002/cam4.3138)
Supplement: Supplementary file 1 — Table S1‐S4 [file CAM4-9-6933-s001.docx]

**Supplemental Table 1 – Population-based health services databases used to derive high-intensity end-of-life care measures**

| **Database** | **Data Elements** | **Description** | **Initiation Year** |
| --- | --- | --- | --- |
| DAD | Inpatient hospitalizations | One record per hospital admission including chart-abstracted demographic, clinical and outcome data. | 1988 |
| NACRS | ED visits | Demographic, clinical and disposition data. | 2000 |
| OHIP | Physician claims | Claims for services billed by fee-for-service Ontario physicians. Physicians under alternative funding plans are also required to submit shadow claims, ensuring capture of nearly all physician encounters. | 1991 |

ALR – Activity Level Reporting; DAD – Discharge Abstract Database; ED – emergency department; NACRS – National Ambulatory Care Reporting System; OHIP – Ontario Health Insurance Plan Claims Database

**Supplemental Table 2. Univariate and multivariable predictors of event-free survival, with stage added to multivariable model**

|  | **Univariate** | | **Multivariable** | |
| --- | --- | --- | --- | --- |
|  | HR (95CI) | p value | HR (95CI) | p value |
| Age (per year) | 1.0 (1.0-1.1) | 0.25 | - | - |
| Sex |  |  |  |  |
| Male | Ref | Ref | - | - |
| Female | 0.9 (0.7-1.2) | 0.46 | - | - |
| Time period |  |  |  |  |
| Early (1992-1998) | Ref | Ref | - | - |
| Middle (1999-2005) | 1.0 (0.7-1.4) | 0.85 | - | - |
| Late (2006-2011) | 1.0 (0.7-1.5) | 0.89 | - | - |
| Neighborhood income quintile |  |  |  |  |
| Q1 (lowest) | 1.0 (0.6-1.5) | 0.87 | - | - |
| Q2 | 0.8 (0.6-1.3) | 0.40 | - | - |
| Q3 | 0.7 (0.5-1.1) | 0.13 | - | - |
| Q4 | 0.7 (0.5-1.1) | 0.16 | - | - |
| Q5 (highest) | Ref | Ref | - | - |
| Rurality |  |  |  |  |
| Urban | Ref | Ref | - | - |
| Rural | 0.9 (0.6-1.3) | 0.51 | - | - |
| Histology |  |  |  |  |
| Nodular sclerosis | Ref | Ref | Ref | Ref |
| Other | **0.6 (0.4-1.0)** | **0.05** | **0.6 (0.3-0.9)** | **0.02** |
| Stage |  |  |  |  |
| I | Ref | Ref | Ref | Ref |
| II | 1.1 (0.6-2.0) | 0.69 | 1.1 (0.6-2.0) | 0.71 |
| III | 1.3 (0.7-2.5) | 0.36 | 1.2 (0.6-2.4) | 0.51 |
| IV | 1.6 (0.8-3.1) | 0.15 | 1.4 (0.7-2.8) | 0.34 |
| B symptoms |  |  |  |  |
| No | Ref | Ref | Ref | Ref |
| Yes | **1.6 (1.2-2.1)** | **0.0007** | **1.7 (1.3-2.3)** | **0.0004** |
| Treatment modality |  |  |  |  |
| Chemotherapy only | Ref | Ref | Ref | Ref |
| Radiation only | **1.6 (1.0-2.5)** | **0.05** | **2.2 (1.3-3.7)** | **0.001** |
| Combined modality | **0.7 (0.5-1.0)** | **0.03** | **0.7 (0.5-1.0)** | **0.04** |
| Locus of Care |  |  |  |  |
| Paediatric | Ref | Ref | Ref | Ref |
| RCC | 0.9 (0.7-1.3) | 0.59 | 0.8 (0.6-1.2) | 0.36 |
| Adult community centre | 1.0 (0.7-1.6) | 0.84 | 1.1 (0.7-1.8) | 0.59 |

HR – hazard ratio; RCC – regional cancer centre

**Supplemental Table 3. Univariate and multivariable predictors of overall survival, with histology and stage added to multivariable model**

|  | **Univariate** | | **Multivariable** | |
| --- | --- | --- | --- | --- |
|  | HR (95CI) | p value | HR (95CI) | p value |
| Age (per year) | 1.0 (0.9-1.1) | 0.63 | - | **-** |
| Sex |  |  |  |  |
| Male | Ref | Ref | - | - |
| Female | 0.7 (0.5-1.1) | 0.12 | - | - |
| Time period |  |  |  |  |
| Early (1992-1998) | Ref | Ref | - | - |
| Middle (1999-2005) | 1.1 (0.6-1.7) | 0.84 | - | - |
| Late (2006-2011) | 0.7 (0.4-1.4) | 0.41 | - | - |
| Neighborhood income quintile |  |  |  |  |
| Q1 (lowest) | 0.8 (0.2-1.5) | 0.48 | 1.0 (0.5-1.9) | 0.96 |
| Q2 | 0.7 (0.4-1.3) | 0.28 | 0.9 (0.5-1.8) | 0.80 |
| Q3 | 0.5 (0.2-1.0) | 0.05 | 0.6 (0.3-1.2) | 0.14 |
| Q4 | 0.5 (0.3-1.0) | 0.05 | 0.6 (0.3-1.2) | 0.16 |
| Q5 (highest) | Ref | Ref | Ref | Ref |
| Rurality |  |  |  |  |
| Urban | Ref | Ref | - | - |
| Rural | 1.3 (0.7-2.3) | 0.39 | - | - |
| Histology |  |  |  |  |
| Nodular sclerosis | Ref | Ref | Ref | Ref |
| Other | 1.2 (0.7-2.2) | 0.48 | 1.0 (0.5-2.0) | 0.99 |
| Stage |  |  |  |  |
| I | Ref | Ref | Ref | Ref |
| II | 0.9 (0.4-2.4) | 0.9 | 0.8 (0.3-2.1) | 0.69 |
| III | 1.6 (0.6-4.4) | 0.33 | 1.3 (0.5-3.5) | 0.64 |
| IV | 1.8 (0.6-4.9) | 0.28 | 1.3 (0.5-4.0) | 0.59 |
| B symptoms |  |  |  |  |
| No | Ref | Ref | Ref | Ref |
| Yes | **2.2 (1.4-3.5)** | **0.0008** | **2.0 (1.2-3.2)** | **0.005** |
| Treatment modality |  |  |  |  |
| Chemotherapy only | Ref | Ref | - | - |
| Radiation only | 0.8 (0.4-2.0) | 0.67 | - | - |
| Combined modality | 0.8 (0.5-1.2) | 0.23 | - | - |
| Locus of Care |  |  |  |  |
| Paediatric | Ref | Ref | Ref | Ref |
| RCC | 0.9 (0.5-1.5) | 0.65 | 1.2 (0.6-2.1) | 0.62 |
| Adult community centre | 0.9 (0.5-1.9) | 0.84 | 1.3 (0.6-2.7) | 0.51 |

HR – hazard ratio; CI – confidence interval; RCC – regional cancer centre

**Supplemental Table 4. Cumulative incidence at 25 years from diagnosis of various late effects and of liveborn birth, by locus of care**

|  | Paediatric Centre | Adult Centre | p value |
| --- | --- | --- | --- |
| Second malignant neoplasm | 8.4%±3.1% | 5.7%±1.2% | 0.12 |
| Congestive heart failure | 1.9%±1.3% | 3.5%±1.2% | 0.77 |
| Major cardiovascular event | 1.9%±1.4% | 7.7%±2.2% | 0.12 |
| Liveborn birth | 69.0%±10.2% | 60.5%±3.3% | 0.07 |
